# The Influence of Water Sorption of Dental Light-Cured Composites on Shrinkage Stress

**DOI:** 10.3390/ma10101142

**Published:** 2017-09-28

**Authors:** Kinga Bociong, Agata Szczesio, Krzysztof Sokolowski, Monika Domarecka, Jerzy Sokolowski, Michal Krasowski, Monika Lukomska-Szymanska

**Affiliations:** 1 University Laboratory of Materials Research, Medical University of Lodz, 251 Pomorska St., 92-213 Lodz, Poland; kinga.bociong@umed.lodz.pl (K.B.); agata.szczesio@umed.lodz.pl (A.S.); michal.krasowski@umed.lodz.pl (M.K.); 2 Department of Restorative Dentistry, Medical University of Lodz, 251 Pomorska St., 92-213 Lodz, Poland; krzysztof.sokolowski@umed.lodz.pl; 3 Department of General Dentistry, Medical University of Lodz, 251 Pomorska St., 92-213 Lodz, Poland; monika.domarecka@umed.lodz.pl (M.D.); jerzy.sokolowski@umed.lodz.pl (J.S.)

**Keywords:** dental composites, shrinkage stress, water sorption, photoelastic investigation

## Abstract

The contraction stress generated during the photopolymerization of resin dental composites is the major disadvantage. The water sorption in the oral environment should counteract the contraction stress. The purpose was to evaluate the influence of the water sorption of composite materials on polymerization shrinkage stress generated at the restoration-tooth interface. The following materials were tested: Filtek Ultimate, Gradia Direct LoFlo, Heliomolar Flow, Tetric EvoCeram, Tetric EvoCeram Bulk Fill, Tetric EvoFlow, Tetric EvoFlow Bulk Fill, X-tra Base, Venus BulkFil, and Ceram.X One. The shrinkage stress was measured immediately after curing and after: 0.5 h, 24 h, 72 h, 96 h, 168 h, 240 h, 336 h, 504 h, 672 h, and 1344 h by means of photoelastic study. Moreover, water sorption and solubility were evaluated. Material samples were weighted on scale in time intervals to measure the water absorbency and the dynamic of this process. The tested materials during polymerization generated shrinkage stresses ranging from 6.3 MPa to 12.5 MPa. Upon water conditioning (56 days), the decrease in shrinkage strain (not less than 48%) was observed. The decrease in value stress in time is material-dependent.

## 1. Introduction

An integral feature of all currently available resin-based restorative materials is polymerization shrinkage [1]. Teeth undergo consistent chewing and biting loads. However, during restorative procedures new additional stress appears in the tooth structure, increasing the overall stress levels [2]. As a result of deformation or even cracks in the tooth structure, the damage of the adhesive bond, secondary caries, post-operative sensitivity, and marginal discoloration might be found [3,4]. The dental materials are constantly immersed in saliva, and therefore sorption and solubility occur [5,6]. The water diffuses into the material and causes a gradual expansion and volume increase. This phenomenon should counteract the contraction stress [7,8]. The shrinkage stress of the composite and the techniques employed to characterize the stress development have been investigated for several decades. Various devices and techniques (i.e., micro-leakage, the Bioman shrinkage-stress instrument, finite element analysis, and three-dimensional micro-CT data) have been used for measuring the polymerization shrinkage in terms of volumetric and linear shrinkage [9,10,11,12]. Although there are many articles on the shrinkage stress of dental composites [13,14,15], the in-depth study on the relationship between water sorption and the shrinkage stress generated during curing is missing. The water absorption of resin matrix materials can have a significant effect on material dimensions and cause radial pressure [16]. Versluis et al. [2] digitized restored teeth with an optical scanner and analyzed to determine the deformation patterns. The study showed that polymerization shrinkage deformation was compensated by hygroscopic expansion within 4 weeks in teeth restored with a hydrophobic resin composite, while the hydrophilic restorative material over-compensated polymerization shrinkage within 1 week causing tooth expansion. Bowen et al. [17] showed that dental composite could be formulated to have sufficient hygroscopic expansion to compensate polymerization shrinkage. Researchers reported that dental resins bis-GMA/TEGMA and urethane dimethacrylate-based were fully relieved by water sorption. In some cases hygroscopic expansion caused the new “expansion stress”. Huang et al. [18] studied the effect of water sorption on the extent of marginal gap reduction in deferent types of dental materials. The thin ring-slitting method was used to compare the residual stress generated within composite materials with varying hydrophilicity upon wet and dry aging. The residual shrinkage stresses in dental composites could be reversed during water aging. The effect was directly related to hydrophilic properties of dental composites [19]. 

The hygroscopic expansion of materials that are prone to water uptake can exceed the amount of polymerization shrinkage [2,20,21,22]. Such an over-compensation could generate internal expansion stress, endangering the restored tooth integrity. The amount of hygroscopic expansion and thus its clinical consequences may vary with material characteristics. 

The purpose of this study was to evaluate the influence of water sorption of composite materials on polymerization shrinkage stress generated at the restoration-tooth interface. 

The null hypotheses was: There is no difference in the final magnitude and the dynamic of hygroscopic expansion between the dental materials.

## 2. Results

### 2.1. Absorbency Dynamic Study

**Figure 1:**
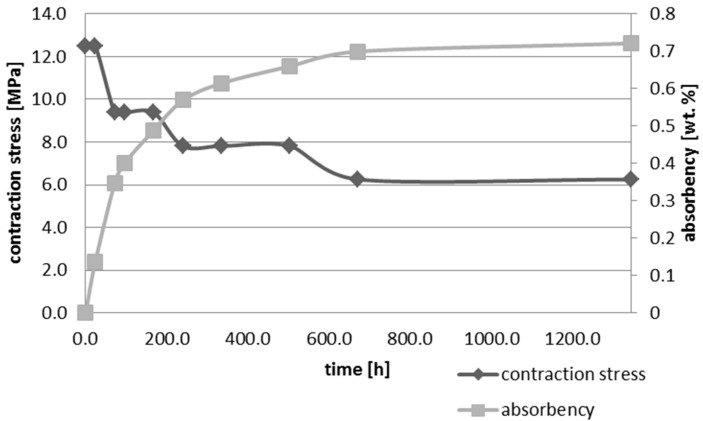
The influence of water sorption (56 days water immersion) on absorbency and contraction stress generated during photopolymerization of Ceram.X One.

**Figure 2:**
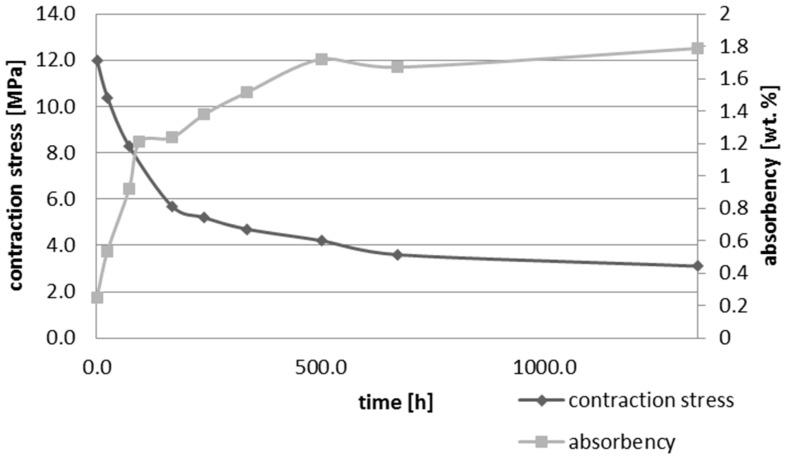
The influence of water sorption (28 days water immersion) on absorbency and contraction stress generated during photopolymerization of Grandia Direct LoFlo.

**Figure 3:**
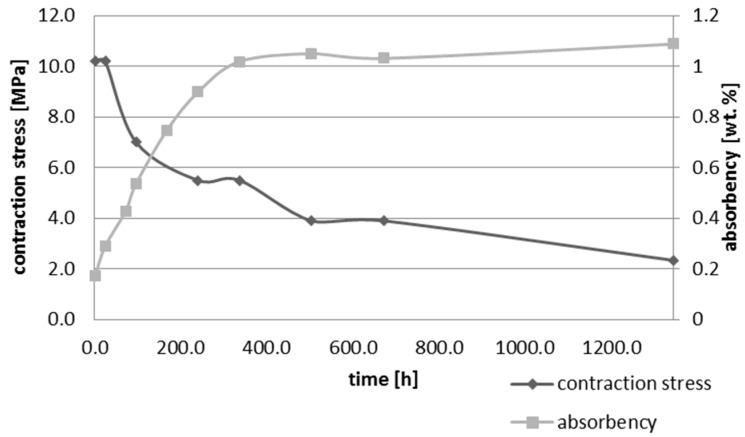
The influence of water sorption (28 days water immersion) on absorbency and contraction stress generated during photopolymerization of Filtek Ultimate.

**Figure 4:**
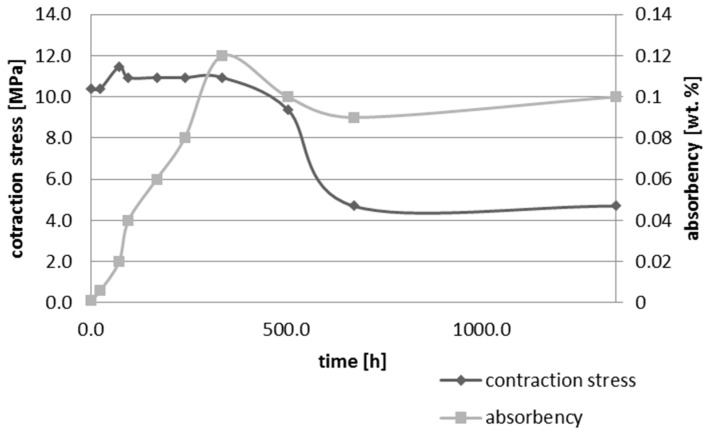
The influence of water sorption (28 days water immersion) on absorbency and contraction stress generated during photopolymerization of X-tra base.

**Figure 5:**
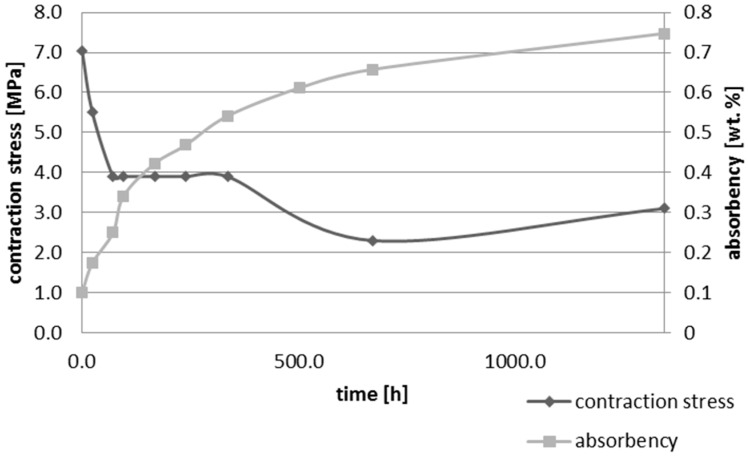
The influence of water sorption (28 days water immersion) on absorbency and contraction stress generated during photopolymerization of Tetric EvoCeram.

**Figure 6:**
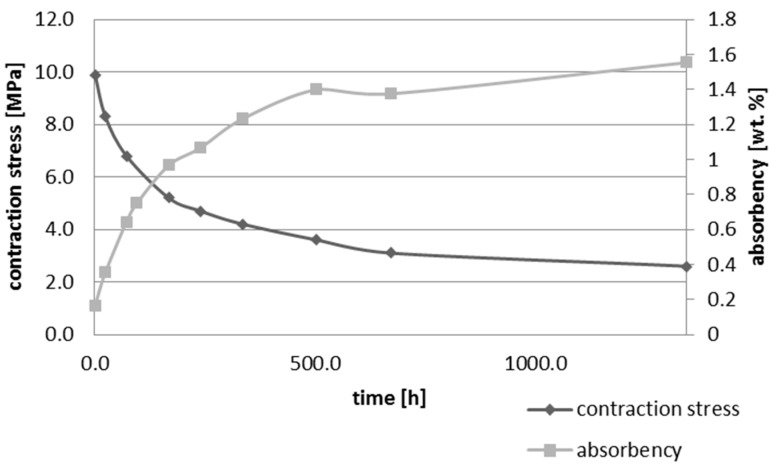
The influence of water sorption (28 days water immersion) on absorbency and contraction stress generated during photopolymerization of Heliomolar Flow.

Upon water immersion, an increase in weight of all tested materials was observed. Water absorbency and contraction stress mean values were presented in Figure 1, Figure 2, Figure 3, Figure 4, Figure 5 and Figure 6. The highest value of absorbency after 56 days (1344 h) was observed for Gradia Direct LoFlo and Heliomolar Flow and the lowest for X-tra Base. Other materials exhibited absorbency (weight %) of 0.6–1.0. 

### 2.2. Water Sorption and Solubility

Mean values of water sorption and solubility were presented in Table 1. Gradia Direct LoFlo and Heliomolar Flow had also the highest values of water sorption, while X-tra Base had the lowest value of water sorption. Other materials exhibited sorption of 10.6–27.1 µg/mm^3^. 

### 2.3. Photoelastic Study

**Figure 7:**
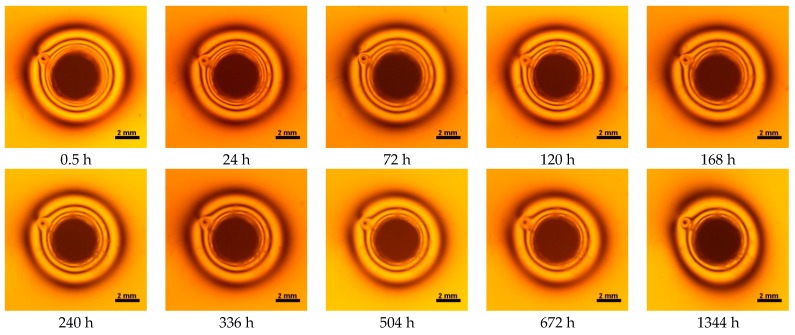
Isochromes in epoxy plate around Ceram.X One restoration before and after water storage 0.5–1344 h.

**Figure 8:**
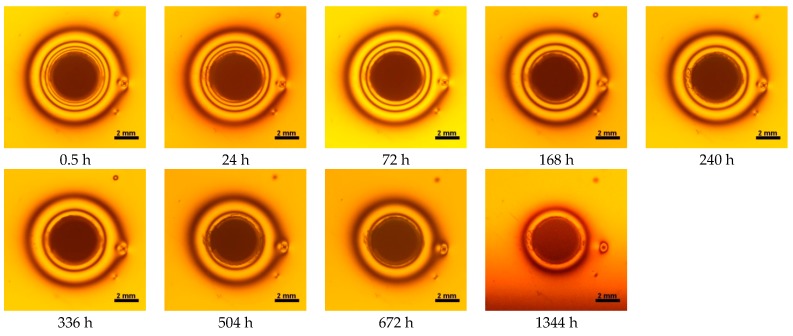
Isochromes in epoxy plate around Gradia Direct LoFlo restoration. Images acquired in polarized light with parallel polarization facets before and after water storage 0.5–1344 h.

**Figure 9:**
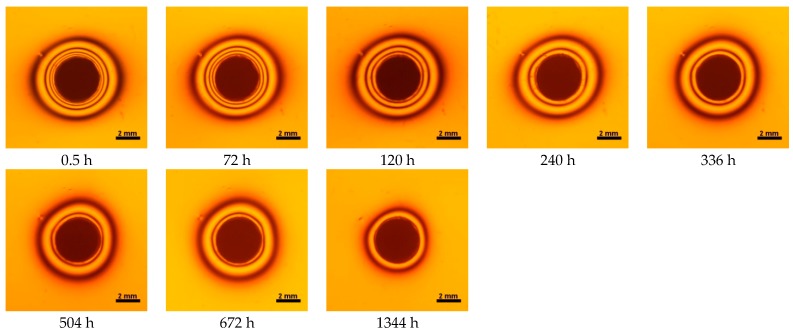
Isochromes in epoxy plate around Filtek Ultimate restoration before and after water storage 0.5–1344 h.

**Figure 10:**
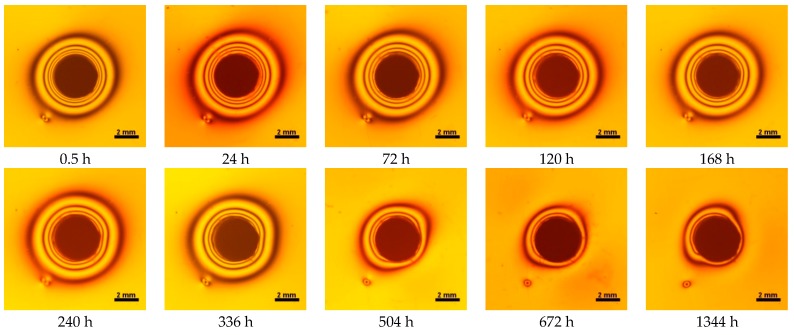
Isochromes in epoxy plate around X-tra base restoration before and after water storage 0.5–1344 h.

**Figure 11:**
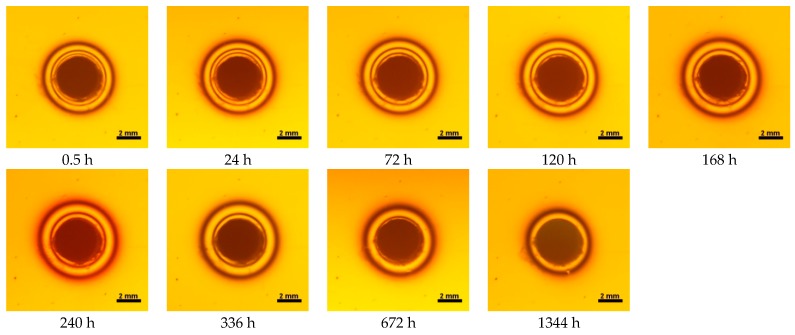
Isochromes in epoxy plate around Tetric EvoCeram restoration before and after water storage 0.5–1344 h.

**Figure 12:**
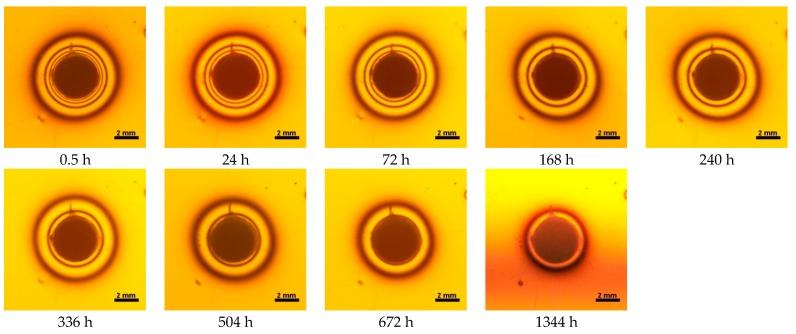
Isochromes in epoxy plate around Heliomolar Flow restoration before and after water storage 0.5–1344 h.

Contraction stress was observed for all selected dental materials. The contraction stress was reduced significantly due to the hygroscopic expansion of composites (Figure 7, Figure 8, Figure 9, Figure 10, Figure 11 and Figure 12). Water sorption and contraction stress mean values were presented in Figure 1, Figure 2, Figure 3, Figure 4, Figure 5 and Figure 6. It was found that Ceram.X One and Gradia Direct LoFlo exhibited the highest contraction stress of ~12 MPa (Figure 1 and Figure 2). The lowest value of contraction stress of ~6 MPa was observed for Tetric EvoCeram Bulk Fill (Table 1). The greatest reduction in contraction stress (~89%) after 56 days, due to hygroscopic expansion of composites, was found for Tetric EvoCeram (Figure 5 and Figure 11). Over 70% reduction in shrinkage stress was observed for Gradia Direct LoFlo, Filtek Ultimate, and Heliomolar Flow (Figure 2, Figure 3 and Figure 6, respectively).

## 3. Discussion

The contraction stress is generated as a result of polymerization shrinkage [1,23,24] and is a major factor of the tooth-filling bond failures [25]. The restoration is exposed to the oral fluids; therefore, the contraction stress may be partially relieved by the water uptake by composite resin [18]. Until our previous study, [26], it had not been demonstrated that an elasto-optic method could be used to evaluate effect of water sorption on reduction of stresses at the restoration-tooth tissue interface (using epoxy resin plate). However, the in-depth analysis of the shrinkage stress value in various resin-based composite materials after water aging is highly needed.

The contraction stress drop after 56 days of water immersion varied significantly between tested materials. It was apparent from Figure 1, Figure 2, Figure 3, Figure 4, Figure 5, Figure 6, Figure 7, Figure 8, Figure 9, Figure 10 and Figure 11 that the dynamics of expansion also differed significantly. Therefore, the null hypothesis with regard to expansion magnitude was rejected.

The Fickian (type I) diffusion process controls water uptake into polymer matrix [27]. The two major models were developed to describe diffusion in polymers. The “free volume theory” assumes that water penetrates through nanopores without any chemical reaction with polymer chains. In the “interaction theory”, water diffuses through the material binding successively to the hydrophilic groups [28]. Therefore, absorbed water exists in two distinct forms: (1) “unbound water” that occupies free volume between the polymer chains and the nanopores created during polymerization [29]; and (2) “bound water” that is attached to polymer chains via hydrogen bonds [30]. This rapid elution of unbound molecules of water into free volume between the chains and crosslinks correlates with decrease in shrinkage stress (Figure 1, Figure 2, Figure 3, Figure 4, Figure 5, Figure 6, Figure 7, Figure 8, Figure 9, Figure 10, Figure 11 and Figure 12). Further reduction of stress levels results from slow water uptake up to the point of saturation. 

The chemistry and the structure of polymer matrix were the most important factors influencing sorption and solubility of dental composites. The differences in water absorption of polymer network depending on monomer type (TEGDMA > Bis-GMA > UDMA > Bis-EMA) were reported [31]. The present study confirmed these results. The highest value of water sorption and water absorbency by weight % were observed for Heliomolar Flow, Gradia Direct LoFlo, and Filtek Ultimate. The contraction stress reduction was higher than 70% for these materials (Table 1). The present results of water sorption and solubility confirm other studies [32,33,34,35]. The majority of above-mentioned composites contained bis-GMA, TEGDMA, and UDMA (Table 2), the most hydrophilic monomers. Bis-GMA, despite the very strong intermolecular interaction and rigid backbone, exhibited low degree of conversion and was prone to water uptake [36,37]. Furthermore, Gradia Direct LoFlo and Filtek Ultimate had low filler content. Fillers reduced the free volume in polymer matrices, decreasing sorption and solution of dental material [38]. Moreover, these composites contained an organic-modified filler. The organic phase in filler could additionally increase the water sorption, but the main role of this phase was to improve the resin-filler connection. This could explain the high values of the stress reduction due to water sorption.

The highest reduction of contraction stress after 56 days water immersion was observed for Tetric EvoCeram (Table 1). The contraction stress reduction amounted up to 89%. Such a high result was a consequence of chemical nature of the material. Highly hydrophilic monomers i.e., bis-GMA and UDMA, caused the hydroscopic relaxation [22]. Tetric EvoCeram had relatively high loss modulus, which suggested greater ability to relieve energy built up through moderate viscous flow. The additional factor was high filler content, since friction between the particles and the resin matrix were indicated as an important element of energy dissipation during deformation under stress [39,40].

The present study proved that Tetric EvoFlow and Tetric EvoFlow Bulk Fil exhibited the same stress value measured immediately after polymerization and after 56 days of water immersion (Table 1). However, minor changes in material composition had no significant effect on stress.

X-tra base was a dental composite with conventional composition of polymer matrix containing bis-EMA, UDMA, and aliphatic dimethacrylate as diluents. The stress relaxation means amounted up to 55% after 28 days of water storage (Table 1). Next, the lowest water absorbency of ~0.7 wt % was observed. Considering weaker hydrophilic character of bis-EMA and UDMA compared to bis-GMA, such a low value of water sorption was understood. Hydroxyl groups of bis-GMA formed stronger hydrogen bonds with water molecules than urethanes group, that also could explain the low value of water absorbency [22]. The mechanism of X-tra base contraction stress relaxation differed from examined materials (i.e., Filtek Ultimate, Gradia Direct LoFlo, Heliomolar Flow, Tetric EvoCeram) and did not result from water sorption. The dynamic of contraction stress relaxation was also different in comparison to material that absorbed more water, i.e., Gradia Direct LoFlo, Filtek Ultimate, or Heliomolar and bulk-fill materials (Table 1). The stress was significantly relieved after two weeks and still decreased. The reduction in shrinkage stress of flowable bulk-fill material i.e., X-tra base, probably resulted from the content of additives such as pre-polymer stress relievers, polymerization modulators, and modified high-molecular-weight base monomers [41]. 

It was also found that contraction stress generated during photopolymerization of Ceram.X One could be significantly relieved by hydroscopic expansion (Table 1, Figure 1 and Figure 7). The expansion resulted from Ceram.X One composition (high amount of hydrophilic monomers) and morphology of pre-polymerized filler (PPF). The PPF exhibited a high degree of sphericity and distinct microstructure; thus, water could be absorbed effectively due to capillary forces and matching polarities of the filler surfaces and penetrating resin.

## 4. Materials and Methods

The composition of investigated material and bonding systems was presented in Table 2 and Table 3.

### 4.1. Absorbency Dynamic Study

**Figure 13:**
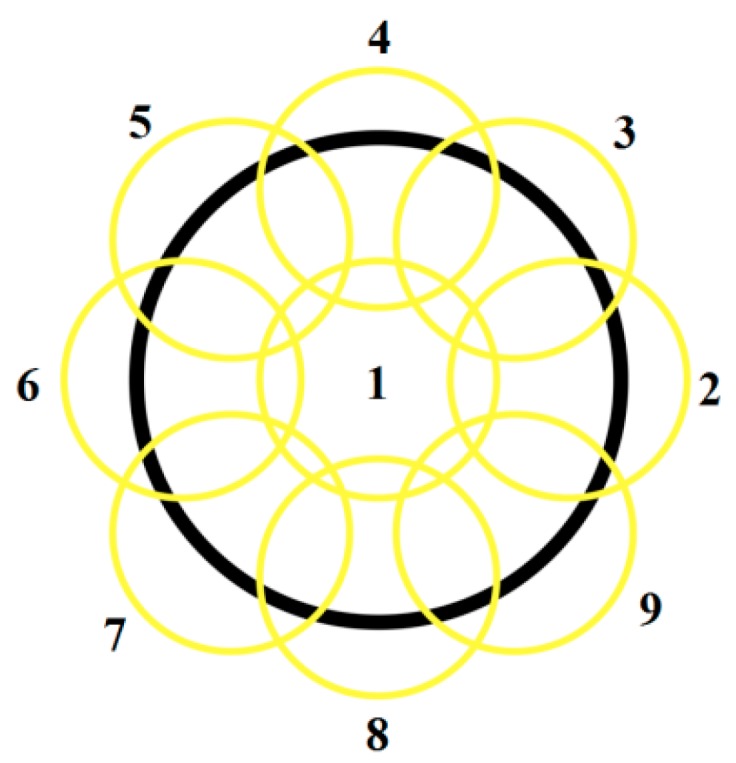
A diagram of partially overlapping zones of curing sample.

In order to determine absorbency dynamic, the samples were prepared using the silicone mold (15 mm in diameter, 1 mm in width). Tested materials were applied in one layer and cured with LED light lamp (Mini L.E.D., Acteon, Norwich, France) in nine zones partially overlapping according to ISO 4049 recommendations (Figure 13). Exposure time was consistent with the manufacturer instructions (Table 2). Direct contact of optical fiber with the sample surface was ensured. 

Five samples were prepared for each dental composite. The samples were weighted (RADWAG AS 160/C/2, Radom, Poland) immediately after preparation and daily for 30 days. The absorbency was calculated according to the Equation (1) [42]:

(1) $$A=\frac{m_{i}-m_{0}}{m_{0}}\cdot 100\%$$

where *A* is the absorbency of water, *m*_0_ is the mass of the sample in dry condition, *m_i_* is the mass of the sample after storage in water for a specified (*i*) period of time.

### 4.2. Water Sorption and Solubility

Water sorption and solubility was investigated according to ISO 4049. Five samples were prepared for each dental composite. The samples were prepared using the silicone mold (15 mm in diameter, 1 mm in width). Tested materials were applied in one layer and cured with LED light lamp (Mini L.E.D., Acteon, France) in nine zones partially overlapping (Figure 13). Exposure time was consistent with the manufacturer instructions (Table 2). Direct contact of optical fiber with the sample surface was ensured. Specimens were placed in a vacuum desiccator (Duran^®^, Mainz, Germany) at a temperature of 37 ± 1 °C for 23 h, transferred to a second desiccator at the temperature of 25 ± 1 °C for 1 h, and then weighed in the balance (RADWAG AS 160/C/2, Radom, Poland). This cycle was repeated until a constant mass was obtained (*m*_1_). Upon stabilization, specimens were immersed in distilled water at a temperature of 37 ± 1 °C for 7 days. Specimens were removed, gently dried with absorbent paper, and weighed again to obtain *m*_2_. Using the same protocol as for *m*_1_, specimens were then reconditioned in the desiccators until a constant mass was obtained (*m*_3_). Water sorption (*W_sp_*) and solubility (*W_sl_*) ratios were calculated for each specimen using the following equations:

(2) $$W_{sp}=100\cdot \frac{m_{2}-m_{1}}{V}$$

(3) $$W_{sl}=100\cdot \frac{m_{1}-m_{3}}{V}$$

### 4.3. Photoelastic Study

In order to evaluate contraction stress, which generates during photopolymerization of resin composites, transparent and photosensitive plates made of epoxy resin (Epidian 53, Organika-Sarzyna SA, Nowa Sarzyna, Poland) were used. The calibrated orifices (3 mm in diameter and 4 mm in thickness) in resin plates were prepared. The circular shape and size of orifices was meant to mimic an average tooth cavity. To obtain higher micromechanical retention, surface of the plates was sandblasted with a 50-μm grain corundum Cobra (Renfert, Hilzingen, Germany). Thus, prepared plates were immersed in distilled water for 3 months to eliminate errors associated with water sorption of resin. Next, dedicated bonding system was applied and cured with Elipar S10 lamp (3M ESPE, Landsberg am Lech, Germany) (Table 3). The orifices were filled with tested material in one layer. Three samples were prepared for each material. The polymerization was performed according to the manufacturer’s instructions (Table 2 and Table 3). Both light curing units (Mini L.E.D and Elipar S10) had an output irradiance of 1250 mW/cm^2^ and 1450 mW/cm^2^, respectively, as stated by the manufacturer. To ensure consistent irradiance values, the light curing units were calibrated with radiometer system (Digital Light Meter 200 from Rolence Enterprice Inc., Taoyuan, Taiwan).

Next, samples were stored in distilled water at room temperature. After selected period of time (30 min, 24 h, 72 h, 120 h, 168 h, 240 h, 336 h, 504 h, 672 h, and 1344 h), the generated strains in the plates were visualized in circular transmission polariscope FL200 (Gunt, Hamburg, Germany). Photoelastic images were registered by digital camera (Canon EOS 5D Mark II/Canon Inc., Tokyo, Japan), both in parallel and perpendicular orientation of filter polarization planes. Met-Ilo computer program (J. Szala, 2012, Poland) was applied to determine the arrangement and the dimension of interference fringes. The analysis of stress and strain was carried out in a two-dimensional state of the stress and three-dimensional state of deformations. The analysis of stress and strain was carried out in a two-dimensional state of the stresses and three-dimensional state of deformations. Additionally, the calculation was conducted following this assumption: the relative change in composite volume caused the extension of composite and the extension of base material being “tooth model” (epoxy resin plate). Accordingly, it was possible to determine the radial and circumferential stresses based on the Equations (4) and (5) given by Timoshenko [43]:

(4) $$\sigma_{r}=\frac{a^{2}\cdot p_{s}}{b^{2}-a^{2}}\cdot \left(\frac{b^{2}}{r^{2}}-1\right)$$

(5) $$\sigma_{\theta }=\frac{a^{2}\cdot p_{s}}{b^{2}-a^{2}}\cdot \left(\frac{b^{2}}{r^{2}}+1\right)$$

where *σ_r_*—the radial stress, *σ_θ_*—the circumferential stress, *p_s_*—the shrinkage stress around composite filling, *a*—the radius of the internal orifices in the plate, *b*—the radius of the largest of isochromatic fringe, and *r*—the radius contained in the region from *a* to *b*.

Upon calculating the shrinkage stress on the circumference of the orifices, the radial and circumferential stresses were determined on the basis of Equations (2) and (3).

## 5. Conclusions

It was shown that an elastooptic method could be used to measure the contraction stress and to demonstrate the effect of water sorption on stress reduction at the restoration-tooth interface.

Moreover, tested resin dental materials generated differentiated contraction stress during photopolymerization. The value of contraction stress, water absorbency, magnitude of reduction, and dynamics of stress change were material-dependent properties.

## Figures and Tables

**Table 1:** Contraction stress before and after 56 days water immersion, contraction stress drop, absorbency, and solubility of tested materials.

Material	Contraction Stress after 0.5 h (MPa)	Contraction Stress after 56 Days of Water Immersion (MPa)	Contraction Stress Drop (%)	Sorption (µg/mm^3^)	Solubility (µg/mm^3^)
Filtek Ultimate	10.2 ± 1.1	2.3 ± 1.1	77	27.1 ± 1.1	2,8 ± 1,5
Gradia Direct LoFlo	12.0 ± 0.9	3.1 ± 0.1	74	35.0 ± 0.9	2.1 ± 0.5
Heliomolar Flow	9.9 ± 0.9	2.6 ± 0.9	74	34.6 ± 0.3	2.6 ± 0.4
Tetric EvoCeram	7.0 ± 1.1	0.8 ± 0.2	89	19.5 ± 1.1	1.1 ± 0.1
Tetric EvoCeram Bulk Fill	6.3 ± 0.3	3.1 ± 0.1	51	17.7 ± 1.0	1.1 ± 0.8
Tetric EvoFlow	9.4 ± 0.5	4.7 ± 0.2	50	20.2 ± 0.3	2.4 ± 0.2
Tetric EvoFlow Bulk Fill	9.4 ± 0.3	4.7 ± 0.2	50	10.6 ± 0.1	1.7 ± 0.3
X-tra Base	10.4 ± 0.9	4.7 ± 0.2	55	6.6 ± 2.3	0.2 ± 0.1
Venus BulkFil	8.1 ± 1.1	4.2 ± 0.9	48	17.4 ± 0.6	2.0 ± 0.6
Ceram.X one	12.5 ± 0.4	6.3 ± 0.2	50	15.9 ± 1.2	0.5 ± 0.1

**Table 2:** The composition of investigated materials.

Material	Manufacturer (Country)	Composition	Curing Time (s)	Type
Filtek Ultimate	3 M ESPE (USA)	bis-GMA, UDMA, TEGDMA, bis-EMA, PEGDMA, silica, zirconia (79 wt %)	10	Nanocomposite
Gradia Direct LoFlo	GC (Japan)	UDMA, dimethacrylate component (trade secret), fluoro-alumino-silicate glass filler, HDR pre-polymerized fillers (40 wt %)	10	Microhybrid
Heliomolar Flow	Ivoclar Vivadent (Germany)	bis-GMA, UDMA, TEGDMA, highly dispersed silicon dioxide, prepolymer, ytterbium trifluoride (51 wt %)	20	flowable resin composite
Tetric EvoCeram	Ivoclar Vivadent	bis-GMA, UDMA, ethoxylated bis-EMA, barium glass, ytterbium trifluoride, spherical mixed oxide, acyl phosphine oxide (75 wt %)	10	Nanohybrid
Tetric EvoCeram Bulk Fill	Ivoclar Vivadent	bis-GMA, UDMA, barium glass, ytterbium trifluoride, mixed oxide, prepolimerized filler, acyl phosphine oxide (80 wt %)	10	Nanohybrid
Tetric EvoFlow	Ivoclar Vivadent	Bis-GMA, UDMA, decanediol dimethacrylate, barium glass, ytterbium trifluoride, silica, mixed oxide, acyl phosphine oxide (62 wt %)	10	nanohybrid flowable composite
Tetric EvoFlow Bulk Fill	Ivoclar Vivadent	bis-GMA EBADMA, highly reactive patented Ivocerin light initiator, composite filler (62 wt %)	10	bulkfill, nanohybrid
X-tra Base	VOCO (Germany)	bis-EMA, aliphatic dimethacrylate, UDMA, 75 wt % filler loading	10	bulkfill, microhybrid
Venus Bulk Fill	Heraeus (Japan)	UDMA, EBADMA (bis-EMA), ethyl-4-dimethylaminobenzoate, BHT barium-alumino-fluoro-silicate glasses, ytterbiumtrifluoride, silicon dioxide (65 wt %)	20	flowable, low-shrinkage composite/bulkfill
Ceram.X one	Dentsply (USA)	dimethacrylate resin, methacrylate modified polysiloxane, ethyl-4(dimethylamino)benzoate, barium-aluminium-borosilicate glass, methacrylate functionalised silicon dioxide nano filler (76 wt %)	20	nanocomposite

Bis-GMA—bisphenol A glycol dimethacrylate; UDMA—urethane dimethacrylate; bis-EMA—bisphenol A ethoxylateddimethacrylate; TGDMA—triethyleneglycol dimethacrylate PEGDMA—polyethyleneglycol dimethacrylate; HDR—high density radiopaque; BHT—butylated hydroxy toluene; TCB—tetracarboxylic acid-hydroxyethylmethacrylate-ester.

**Table 3:** The composition of bonding systems.

Material	Manufacturer (Country)	Composition	Curing Time (s)	Indicated Composite
Easy Bond	3 M ESPE (USA)	bis-GMA, HEMA, water, ethanol, phosphoric acid 6-methacryloxy-hexylesters, silane treated silica, copolymer of acrylic and itaconic acid, (dimetylamino)ethyl methacrylate	10	Filtek Ultimate
G-Bond	GC (Japan)	4-META, UDMA, TEGDMA, acetone	10	Gradia Direct LoFlo
AdheSE^®^ One F	Ivoclar Vivadent (Germany)	bis-acrylamide derivative, bis-methacrylamide dihydrogenphosphate, amino acid acrylamide, hydroxyalkyl methacrylamide, water, stabilisers, initiators	10	Heliomolar, Tetric EvoCeram, EvoFlow
OptiBond	Kerr (USA)	GPDM, mono- and difunctional methacrylate monomers, water, acetone, ethanol, nanofillers, camphorquinone	10	X-tra base
iBOND^®^ Self Etch	Heraeus (Japan)	4-META, UDMA, glutaraldehyde, acetone, water, photoinitiators, stabilizers	20	Venus Bulk Fill
XP Bond	Dentsply (USA)	TCB, PENTA, UDMA, TEGDMA, HEMA, butylated benzenediol (stabilizer), Ethyl-4-dimethylaminobenzoate; Camphorquinone; Functionalized amorphous silica; t-butanol	10	Ceram.X One

Bis-GMA—bisphenol A glycol dimethacrylate; UDMA—urethane dimethacrylate; bis-EMA—bisphenol A ethoxylateddimethacrylate; TGDMA—triethyleneglycol dimethacrylate PEGDMA—polyethyleneglycol dimethacrylate; HDR—high density radiopaque; BHT—butylated hydroxy toluene; TCB—tetracarboxylic acid-hydroxyethylmethacrylate-ester, GPDM—lycerol phosphate dimethacrylate, PENTA—Phosphoric acid modified acrylate resin, HEMA—2-hydroxyethylmethacrylate.
